# Prevalence and distribution of non-typhoidal *Salmonella enterica* serogroups and serovars isolated from normally sterile sites: A global systematic review

**DOI:** 10.1017/S0950268823001693

**Published:** 2023-10-18

**Authors:** Nienke N. Hagedoorn, Shruti Murthy, Megan Birkhold, Christian S. Marchello, John A. Crump, the Vacc-iNTS Consortium Collaborators

**Affiliations:** 1 Centre for International Health, University of Otago, Dunedin, New Zealand; 2 Department of Surgery, University of Maryland School of Medicine, Baltimore, Maryland, United States of America

**Keywords:** non-typhoidal *Salmonella*, systematic review, meta-analysis, prevalence, *Salmonella* vaccines

## Abstract

To inform coverage by potential vaccines, we aimed to systematically review evidence on the prevalence and distribution of non-typhoidal *Salmonella enterica* serogroups and serovars. We searched four databases from inception through 4 June 2021. Articles were included that reported at least one non-typhoidal *S. enterica* strain by serogroup or serovar isolated from a normally sterile site. Of serogrouped isolates, we pooled the prevalence of serogroup O:4, serogroup O:9, and other serogroups using random-effects meta-analyses. Of serotyped isolates, we pooled the prevalence of *Salmonella* Typhimurium (member of serogroup O:4), *Salmonella* Enteritidis (member of serogroup O:9), and other serovars. Of 82 studies yielding 24,253 serogrouped isolates, the pooled prevalence (95% CI) was 44.6% (36.2%–48.2%) for serogroup O:4, 45.5% (37.0%–49.1%) for serogroup O:9, and 9.9% (6.1%–13.3%) for other serogroups. Of serotyped isolates, the pooled prevalence (95%CI) was 36.8% (29.9%–44.0%) for *Salmonella* Typhimurium, 37.8% (33.2%–42.4%) for *Salmonella* Enteritidis, and 18.4% (11.4%–22.9%) for other serovars. Of global serogrouped non-typhoidal *Salmonella* isolates from normally sterile sites, serogroup O:4 and O:9 together accounted for 90%, and among serotyped isolates, serovars Typhimurium and Enteritidis together accounted for 75%. Vaccine development strategies covering serogroups O:4 and O:9, or serovars Typhimurium and Enteritidis, have the potential to prevent the majority of non-typhoidal *Salmonella* invasive disease.

## Key results

- Non-typhoidal *Salmonella* invasive infections confirmed by culture of normally sterile sites have a case fatality ratio of 15%.
- Vaccine products for human non-typhoidal *Salmonella* disease are currently in development.
- To inform coverage by potential vaccines, we estimate global non-typhoidal *Salmonella* serogroup and serovar coverage by region and by age groups.
- Serogroups O:4 and O:9 account for 90% of isolates of non-typhoidal *Salmonella enterica* from normally sterile sites.
- Serovars Typhimurium and Enteritidis, members of serogroup O:4 and O:9, respectively,account for 75% of isolates of non-typhoidal *Salmonella enterica* from normally sterile sites.

## Introduction

Worldwide, bacterial infections caused by *Salmonella enterica* are responsible for substantial illness and death [1]. Invasive *Salmonella* disease can be grouped into that caused by the typhoidal *Salmonella* serovars Typhi, Paratyphi A, Paratyphi B, and Paratyphi C, and that caused by the non-typhoidal *Salmonella* serovars [2]. Non-typhoidal *Salmonella* (NTS) is classified into 67 serogroups based on the O antigen, the polysaccharide component of the lipopolysaccharide of the outer membrane, and can be further classified into more than 2,500 serovars based on the flagellar H antigen [3].

NTS serovars usually have their reservoirs in animals. Humans are often infected by the consumption of contaminated foods of animal origin through contaminated water, or by the faecal-oral route during contact with reservoir species or their environments [4–6]. However, it has been suggested humans could be a reservoir of NTS serovars and sequence types (STs) associated with invasive disease in Africa [7, 8]. NTS may cause diarrhoeal disease that is generally self-limiting in healthy adults, whereas invasive disease can develop in the absence of current or recent diarrhoea and carries a case fatality ratio of 15% [9]. Persons at increased risk for invasive disease include those with human immunodeficiency virus (HIV) infection, current or recent malaria, and children with malnutrition [1, 10, 11]. We recently performed a global systematic review on the complications and mortality of NTS invasive disease [9], and reported that *Salmonella* Typhimurium and *Salmonella* Enteritidis, members of serogroups O:4 and O:9, respectively, were the most abundant serotypes collectively accounting for 78% to 94% of isolates [9, 12]. Furthermore, *Salmonella* Typhimurium, *Salmonella* Enteritidis, *Salmonella* Infantis, *Salmonella* Newport, and monophasic *Salmonella* Typhimurium [1, 4, 5, 12] have been reported to be the top five serovars in human surveillance data from the United States of America and the European Union, but their distribution might differ in other regions [13, 14]. In addition below the serovar level, specific STs including *Salmonella* Typhimurium ST313 and clades of *Salmonella* Enteritidis ST11 predominate as causes of non-typhoidal *Salmonella* invasive disease in sub-Saharan African countries and are associated with antimicrobial-resistant infections [15–18].

Vaccine candidates for NTS invasive disease in preclinical or early clinical phase of development have been described in detail by Baliban *et al.* [19] and others [20–27] (see Table 1). To assess the potential serogroup and serovar coverage of current and future vaccine candidates, we extended our previous systematic review to estimate the prevalence and distribution of non-typhoidal *Salmonella enterica* serogroups and serovars isolated from normally sterile sites.

**Table 1.** Non-typhoidal *Salmonella enterica* invasive disease vaccines in development and estimated isolate coverage

Type of vaccine	Vaccine antigen Serovar (serogroup)	Estimated isolate coverage in non-typhoidal *Salmonella* based on estimated crude prevalence	
		Serogroup: assuming cross-protection for serovars within the same serogroup	Serovar: coverage at the serovar level without assuming any cross-protection
**Generalised Modules for Membrane Antigens (GMMA)**			
*Salmonella* Typhimurium GMMA, as part of a 2-component *Salmonella* Enteritidis/*Salmonella* Typhimurium GMMA vaccine [25]	*Salmonella* Enteritidis (O:9)	30.5%	27.4%
	*Salmonella* Typhimurium (O:4)	63.3%	59.7%
**Glycoconjugates**			
Non-typhoidal *Salmonella* Core and O-polysaccharide conjugated to the flagellin protein. Trivalent *Salmonella* Conjugate Vaccine [22–24]	*Salmonella* Enteritidis (O:9)	30.5%	27.4%
	*Salmonella* Typhimurium (O:4)	63.3%	59.7%
	*Salmonella* Typhi Vi (O:9)^a^	—^a^	—^a^
**Live-attenuated approaches**			
*Salmonella* Typhimurium strain LH1160 chicken isolate [20]	*Salmonella* Typhimurium (O:4)	63.3%	59.7%
Human *Salmonella* Typhimurium enterocolitis isolate [21]	*Salmonella* Typhimurium (O:4)	63.3%	27.4%

*Source:* Adapted from Baliban *et al.* [19, 26].

a *Salmonella* Typhi not part of this review.

## Methods

### Study design, selection criteria, and search strategy

This study is reported according to the Preferred Reporting Items for Systematic Reviews and Meta Analyses (PRISMA) guidelines [28]. As only published data were used, overview and approval by an institutional review board was not required. The study protocol was registered in the Prospective Register of Systematic Reviews (PROSPERO) on 27 November 2022, and is available online as protocol CRD42022376658 [29].

The study was an extension of the previous global systematic review and meta-analysis described by Marchello *et al.* [9] that reported complications and case fatality ratio among persons with NTS invasive disease. In brief, a literature search was performed in Embase (Ovid), MEDLINE (Ovid), Web of Science (Clarivate), and PubMed from database inception through to 4 June 2021 with the following keywords: non-typhoidal *Salmonella*; specific serovars such as *Salmonella* Typhimurium, and *Salmonella* Enteritidis; and mortality or complications [9]. The search was not restricted by language, country, or date. Primary research articles were included that were peer-reviewed and reported the number of NTS isolates and at least one NTS strain defined by serogroup or serovar, confirmed by culture of samples taken from normally sterile sites (e.g., blood, bone marrow, cerebrospinal fluid, deep tissues, pleural fluid, or synovial fluids). Articles were excluded if they only reported within specific disease groups such as severe malaria or isolates solely from stool or urine samples. Likewise, those that focused on a single *Salmonella* serovar alone, or only on antimicrobial-resistant isolates were not included. The same exclusion process was applied to case reports, case series, policy reports, commentaries, and conference abstracts. In contrast to the original systematic review, studies for this extension were eligible when they reported at least one NTS strain by serogroup or serovar irrespective of whether they detailed the proportion of complications, or deaths.

Search results from each electronic database were downloaded, imported into EndNote X20 (Clarivate, London, UK), and duplicates were subsequently removed. To assess eligibility for the present study, the 291 articles that passed the initial title and abstract review were reviewed again by NNH and SM; a final decision on their eligibility for inclusion was resolved through discussion, or by decision of a third reviewer (JAC).

### Data abstraction

Data were abstracted in Google Forms (Google LLC, USA). Abstracted data included study characteristics such as country, data collection period, United Nations (UN) region [30], and participant age groups, that is, children ≤15 years, adults >15 years, or mixed ages. We recorded the total number of NTS isolates, and the number of isolates for each reported serogroup, or serovar. Isolates were classified as ‘not further identified’ when reported as a mixed group of unspecified serogroups or serovars, or when classified as NTS and were not reported to the serogroup or serovar level. The number of NTS isolates that were not serogrouped or serotyped could not reliably be abstracted. Serovars were grouped into serogroups following the antigenic formulae of *Salmonella* serovars (Supplementary Table S1) [3]. Serogroups were reported according to the current serogroup designation with the historic designation provided in parenthesis at first use, for example, serogroup O:4 (B). Serogroups comprising <10 isolates were combined as ‘other serogroups with <10 isolates.’ Serovars that could not be serogrouped were classified as undesignated. We sought to abstract data on serogroup or serovar by host risk factor, and to analyse the latter when reported with sufficient frequency to make that viable. Next, we recorded the reported serotyping methods including agglutination testing, polymerase chain reaction, multilocus sequence typing, other, or unclear. For articles that reported that ‘standard methods’ were used, we assumed that this indicated agglutination testing, and where the latter were used, we recorded whether the antiserum panel available to the laboratory was reported. As a proxy for level of development, countries were placed into one of two groups based on their income level from the World Bank classification in 2022: lower- and middle-income countries (LMICs), and high-income countries (HICs) [31].

#### Bias assessment

Risk of bias was assessed independently by one of CSM, MB, NNH, or SM, using the same methodology as the primary article (Supplementary Table S2) [9]. We evaluated study design, study setting, patient selection, and microbiology methods. Each question was scored as unknown, low, or high risk. The scores were aggregated to assign each study as having a low, moderate, or high risk of bias. Conflicts were resolved through discussion or by decision of a third author.

### Data analysis

First, the characteristics of the included articles were described. Second, the proportion of serogroups and for serovars were recorded, and third longitudinal trends were explored by grouping articles by decade according to the median year of the reported period of data collection. Fourth, the geographic distribution of serogroups and serovars of all NTS isolate was assessed by UN region and their rank order in prevalence. Lastly, the distribution of serogroups and serovars by HIV infection status of participants was described, assuming that only one isolate was reported per participant. The proportion of serogroups O:4, O:9, and other serogroups, as well as the proportion of *Salmonella* Typhimurium, *Salmonella* Enteritidis, and other serovars, according to HIV infection status of subjects, were compared using the Chi^2^ test.

#### Meta-analysis

For isolates identified to the serogroup level, we pooled the prevalence across serogroup O:4, serogroup O:9, and other serogroups. A meta-analysis in MetaXL version 5.3 (EpiGear International Pty Ltd., Australia) was performed using the DerSimonian-Laird random-effects model for multiple categories with the double arcsine transformation [31]. Subgroup analyses were performed by UN region, income group, and age group. In addition, for isolates identified to the serovar level, their prevalence across *Salmonella* Typhimurium, *Salmonella* Enteritidis, and other serovars was pooled. Heterogeneity across studies was assessed using forest plots, Chi^2^ test, *I^2^* statistic, and Tau2 (τ^2^). A I^2^ value of 0–49% was considered to be indicative of low heterogeneity, 50–74% as moderate, and ≥ 75% as substantial [32]. A *p*-value <0.05 was considered as significant heterogeneity.

In addition, the proportion of NTS isolates that might be covered by current vaccines in development based on the prevalence of serogroups and serovars was estimated (Table 1). While the mechanism and immune correlates of protection of vaccines in development against NTS invasive disease are not known, the potential coverage at both the serogroup and the serovar levels was examined. For potential coverage at the serogroup level, it was assumed that coverage of vaccines would provide cross-protection for all serovars within the same serogroup. Coverage was also explored with the incremental addition of other common serogroups and serovars. Apart from the meta-analysis in MetaXL, other analyses were performed in R version 4.2 (packages: dplyr, forestploter, ggplot2, tidyr).

## Results

### Article characteristics

Of the 291 full-text articles, 82 were included (Supplementary Figure S1; Supplementary Table S3). Of the latter, 31 (37.8%) reported data from Africa, 21 (25.6%) from Asia, 20 (24.4%) from Europe, 9 (10.9%) from the Americas, and 1 (1.2%) from Oceania (Supplementary Table S4, Supplementary Figure S2). UN subregions lacking data included Northern Africa, Central Asia, Micronesia, Melanesia, and Polynesia. Forty-five (54.9%) articles were from LMICs, and 37 (45.1%) articles were from HICs.

Data reported by the included articles were collected from 1941 through 2019 with a median (IQR) duration of data collection of 6 (3–10) years. Of articles, 71 (86.6%) reported data from a hospital setting. The included population was of mixed ages in 36 (43.9%) articles, children in 33 (40.2%) articles, and adults in 13 (15.8%) articles. Samples from normally sterile sites included blood for 80 (97.6%) articles, cerebrospinal fluid for 11 (13.4%), synovial fluid for 7 (8.5%), deep tissue for 5 (6.1%), pleural fluid for 2 (2.4%), bone marrow in 1 (1.2%), and other normally sterile sites in 10 (12.2%).

Overall, 61 (74.4%) articles were assessed as having a high risk for bias, 21 (25.6%) as moderate risk, and none were of low risk (Supplementary Figure S3). Domains with the highest proportion of high risk for bias, included study setting (n = 70, 85.4%), and microbiology methods (n = 61, 74.4%). Reported methods for serotyping were agglutination testing for 49 (59.8%) articles, a combination of methods for 5 (6.1%), 1 (1.2%) multilocus sequence typing, and unclear for 27 (32.9%). Of the 49 articles reporting agglutination testing, 17 (34.7%) provided details of the anti-serum panel used.

### NTS serogroups and serovars

**Figure 1.**
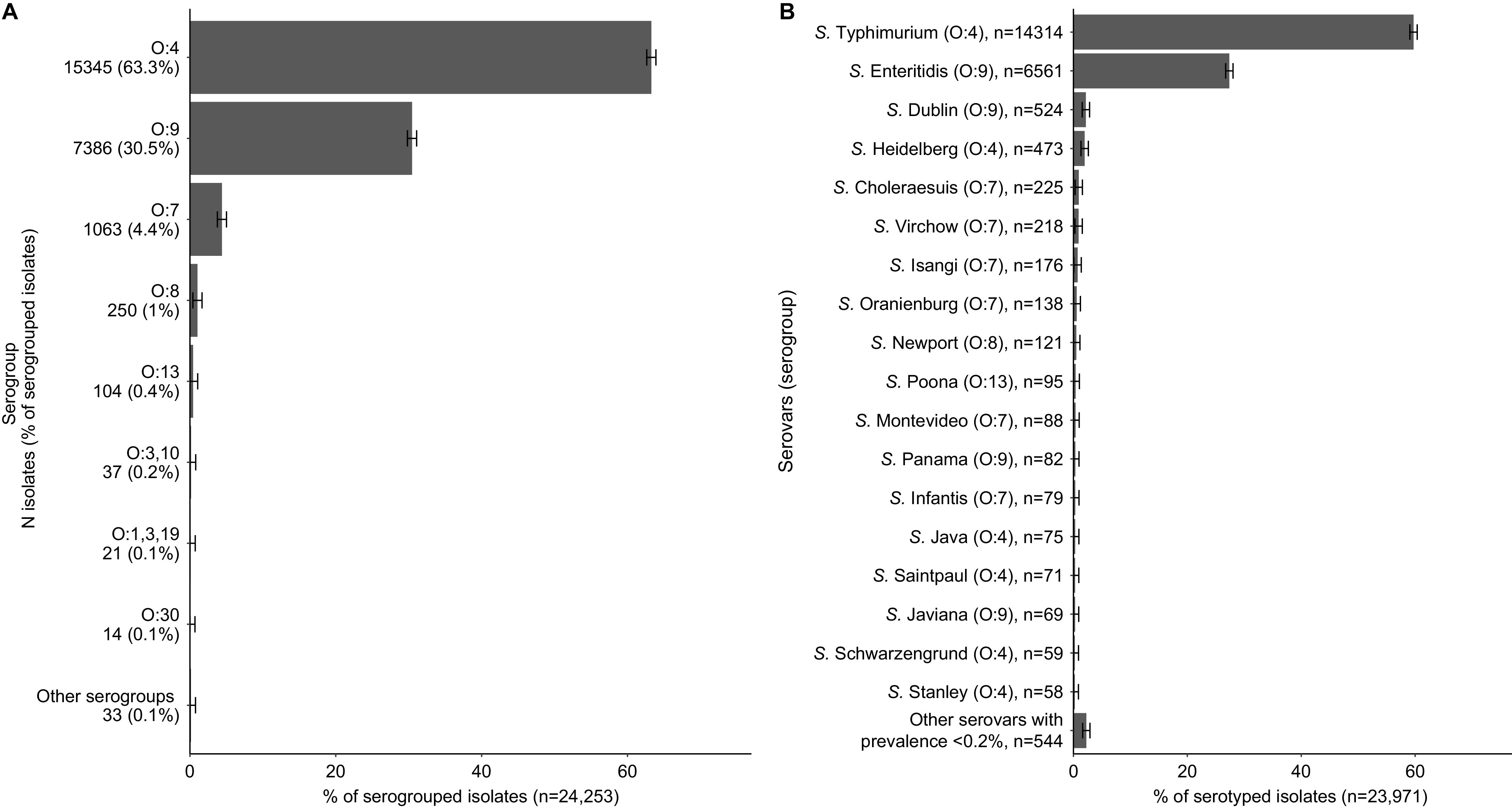
Proportion of non-typhoidal *Salmonella enterica* serogroups (A) and serovars (B) isolated from normally sterile sites, global, 1941–2019. Legend Figure 1: The error bars represent the 95%CI of the proportion. Legend Figure 1a: The complete list of serogroups is provided in Supplementary Table S5. Legend Figure 1b: The complete list of serovars is provided in Supplementary Table S6.

The number of NTS isolates ranged from 1 to 10,139 per study with a median (IQR) of 44 (16–101) isolates per study, yielding a total of 26,277 isolates. Of these isolates, 2,020 (7.7%) were not identified further, 24,253 (92.3%) were serogrouped, and 23,971 (91.2%) serotyped. Four serotyped isolates were undesignated and excluded from the serogrouped isolates. Of the serogrouped isolates, 15,345 (63.3%) were classified as O:4 (B), 7,386 (30.5%) as O:9 (D_1_), 1,063 (4.4%) as O:7 (C_1_), 250 (1.0%) as O:8 (C_2_–C_3_), 104 (0.4%) as O:13 (G). Thirty-three (0.1%) were combined in other serogroups comprising <10 isolates (Figure 1a, Supplementary Table S5).

Of the 23,971 serotyped isolates, 14,317 (54.5%) isolates were serotyped as *Salmonella* Typhimurium, 6,561 (25.0%) as *Salmonella* Enteritidis, 524 (2.2%) as *Salmonella* Dublin, 473 (2.0%) as *Salmonella* Heidelberg, 225 (0.9%) as *Salmonella Choleraesuis*, and 218 (0.9%) as *Salmonella* Virchow (other serovars shown in Figure 1b, Supplementary Table S6). The serogroup distribution by decade is presented in Supplementary Figure S4.

### Geographic distribution of serogroups and serovars

Of serogrouped isolates, 17,350 (71.5%), were from the African region, 3,172 (13.1%) from Europe, 2,645 (10.9%) from the Americas, 1,083 (4.5%) from Asia, and 3 (<0.1%) from Oceania. For Africa, Europe, the Americas, and Asia, serogroups O:4 and O:9 were the two most common (Supplementary Figure S5). The three isolates from Oceania were serogroup O:7. Serogroup O:4 accounted for 73.2% of isolates from the African region and 50.5% of isolates from the Americas. Serogroup O:9 accounted for 57.1% of isolates from the European region. Serogroup O:4 accounted for 38.8% and serogroup O:9 36.7% of isolates from the Asia region. Serogroup O:7 was the third most frequent in Africa, Europe, the Americas, and Asia.

**Figure 2.**
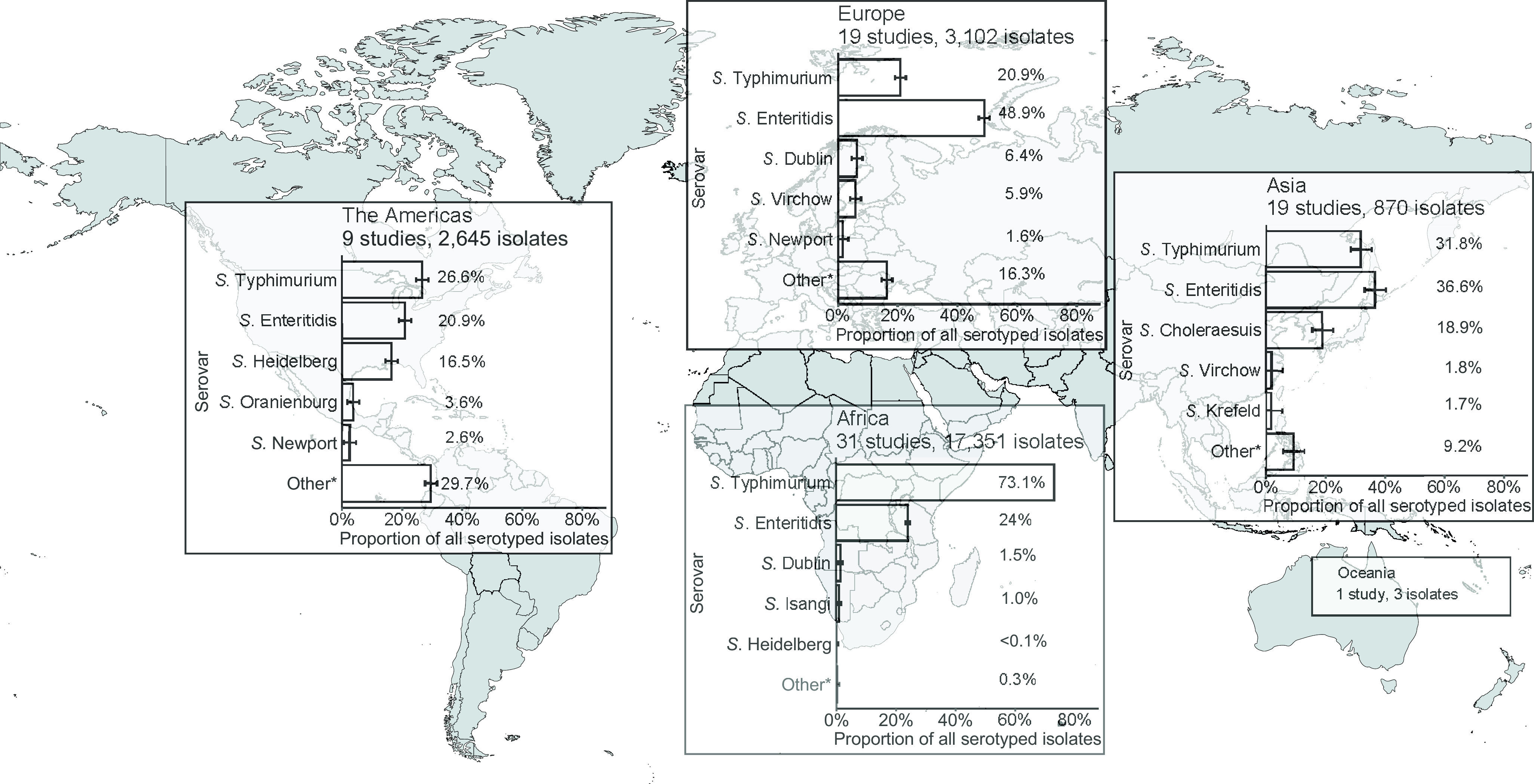
Prevalence of non-typhoidal *Salmonella enterica* serovars from normally sterile sites ranked among top five, by UN region, 1941–2019 (79 articles, 23,971 isolates). Legend Figure 2: The error bars indicate 95%CI of the prevalence. *Others include other serovars that had rank sixth or higher. Data for Oceania is described in the Results section. The global map was downloaded from mapchart.net.

For Africa, Europe, the Americas, and Asia, the serovars *Salmonella* Typhimurium and *Salmonella* Enteritidis were predominant (Figure 2). *Salmonella* Typhimurium accounted for 73.1% of isolates from the African region and 26.6% of isolates from the Americas, and *Salmonella* Enteritidis for 48.9% of those from the European region. *Salmonella* Enteritidis accounted for 36.6% and *Salmonella* Typhimurium for 31.8% of Asian isolates. The third-ranked serovars were *Salmonella* Dublin in Africa and Europe, *Salmonella* Heidelberg in the Americas, and *Salmonella* Choleraesuis in Asia. The proportion of other isolates of serovars ranked sixth and higher was 29.7% in the Americas, 16.3% in Europe, 9.9% in Asia, and 0.3% in Africa.

### Overall pooled prevalence of NTS serogroup, by UN region, income group, and age

**Figure 3.**
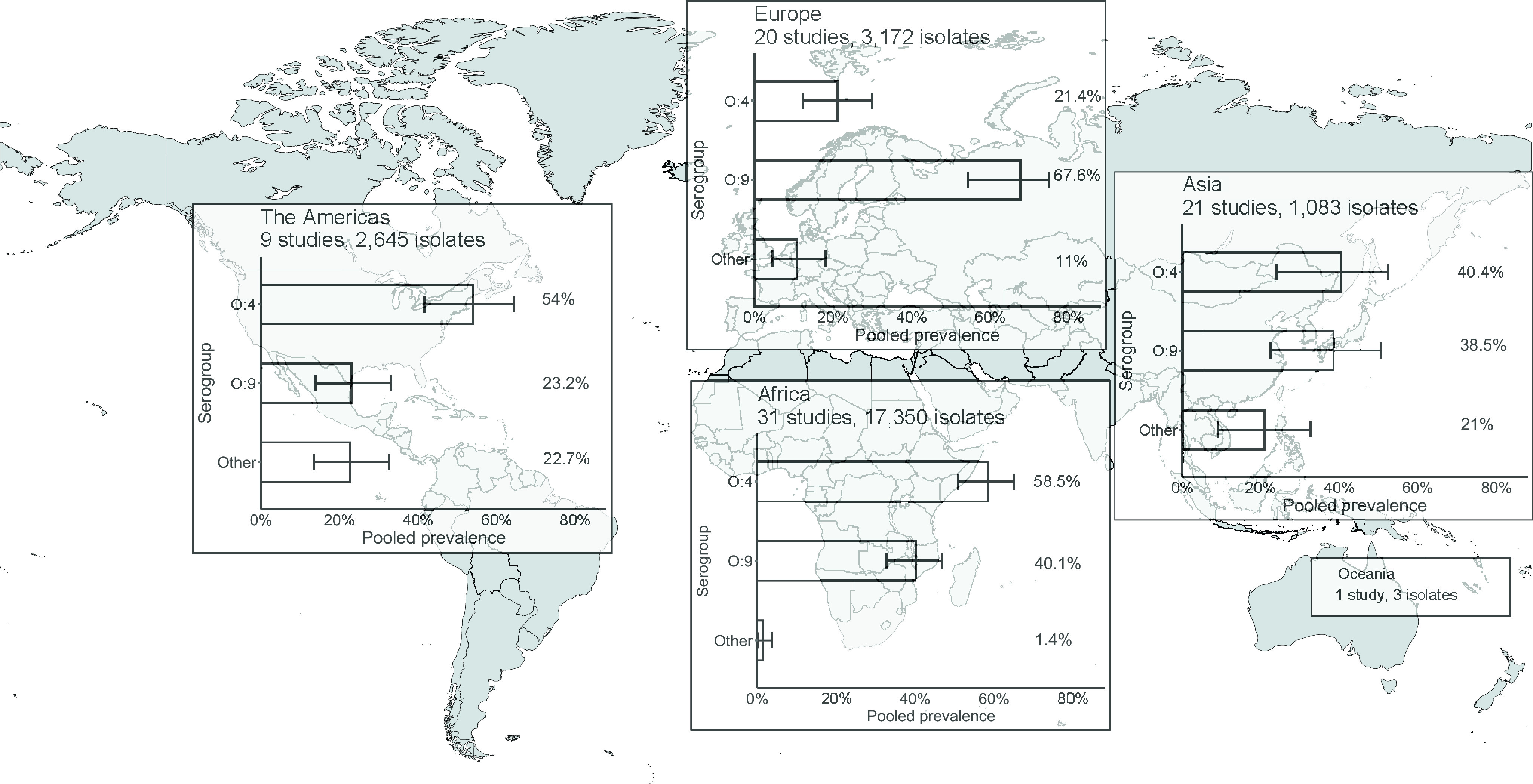
Meta-analysis of prevalence of non-typhoidal *Salmonella enterica* from normally sterile sites for serogroup O:4, serogroup O:9, and other serogroups by UN region, 1941–2019 (82 articles, 24,253 isolates). Legend Figure 3: The error bars indicate 95%CI of pooled prevalence. Data for Oceania was not pooled and is described in the Results. Global map was downloaded from mapchart.net.

In the meta-analysis of the 82 studies reporting 24,253 serogrouped isolates, pooled prevalence (95%CI) was 44.6% (36.2–48.2%) for group O:4, 45% (37.0–49.1%) for group O:9, and 9.9% (6.1–13.3%) for other serogroups (I^2^ = 98.5%, *p* < 0.001) (Supplementary Figure S6). For subgroup analysis by UN region, the pooled prevalence of group O:4 was 58.5% (50.9–65.0%) in Africa (I^2^ = 98.1%, *p* < 0.001), 54.0% (41.7–64.4%) in the Americas (I^2^ = 74.4%, *p* < 0.001), 40.4% (24.2–52.5%) in Asia (I^2^ = 95.4%, *p* < 0.001), and 21.4% (12.4–30.0%) in Europe (I^2^ = 95.3%, *p* < 0.001) (Figure 3, Supplementary Figure S7). For group O:9, the pooled prevalence was 40.1% (32.8–46.9%) in Africa, 23.2% (13.9–33.2%) in the Americas, 38.5% (22.7–50.6%) in Asia, and 67.6% (54.3–74.9%) in Europe. For other serogroups, the pooled prevalence was 1.4% (0.1–3.7%) in Africa, 22.7% (13.5–32.6%) in the Americas, 21.0% (9.3–32.6%) in Asia, and 11.0% (4.7–18.2%) in Europe. The number of isolates from Oceania was insufficient for meta-analysis.

Stratified by income group, the pooled prevalence of serogroup O:4 was 31.4% (22.3–37.8%) in HICs (I^2^ = 96.7%, *p* < 0.001) and 54.7% (45.6–59.2%) in LMICs (I^2^ = 98.0, *p* < 0.001). Prevalence for serogroup O:9 was 51.1% (40.0–56.9%) in HICs and 40.3% (32.1–45.3%) in LMICS. For other serogroups, values were 17.5% (10.8–23.4%) and 5.0% (2.2–8.2%) in high- and low-income groups, respectively. Regarding age distribution of participants, serogroup O:4 accounted for 37.7% (26.4–44.1%) in mixed ages (I^2^ = 99.2%, *p* < 0.001), 51.0% (39.8–57.8%) for children (I^2^ = 95.2%, *p* < 0.001), and 48.3% (28.1–65.1%) for adults (I^2^ = 92.6%, *p* < 0.001) (Supplementary Figure S8). For serogroup O:9, corresponding pooled prevalence was 48.3% (35.6–54.2%) for mixed ages, 43.0% (32.4–50.1%) for children, and 44.1% (24.6%–61.2%) for adults. For other serogroups pooled prevalence was 14.0% (7.3–20.0%) for mixed ages, 6.0% (2.1–10.8%) for children, and 7.5% (0.2–20.4%) for adults. On analysis of isolates at the serovar level, the pooled prevalence was 40.2% (29.5–44.2%) for *Salmonella* Typhimurium, 41.4% (30.5–45.3%) for *Salmonella* Enteritidis, and 18.4% (11.4–22.9%) for other serovars (I^2^ = 99.1%, *p* < 0.001) (Supplementary Figure S9).

### Estimated coverage for vaccine products in development

Assuming cross-protection for serovars within the same serogroup, the estimated coverage of potential vaccine products was 63.3% for serogroup O:4 and 30.5% for serogroup O:9. For vaccines covering both serogroups O:4 and O:9, the coverage was estimated to be 93.8% for isolates classified to the serogroup level (Table 1). The addition of serogroups O:7 and O:8 was estimated to increase coverage by 4.4% and 1.0%, respectively. For protection at serovar level, the estimated coverage was 59.7% for vaccines protecting against *Salmonella* Typhimurium alone and was 27.4% for vaccines protecting against *Salmonella* Enteritidis alone, and estimated coverage against both serovars was 87.1%. The addition of *Salmonella* Dublin, *Salmonella* Heidelberg, or *Salmonella* Choleraesuis would marginally increase coverage by 2.2%, 1.9%, and 0.9%, respectively.

## Discussion

Our global systematic review and meta-analysis of the prevalence and geographic distribution of non-typhoidal *Salmonella enterica* serogroups and serovars isolated from normally sterile sites showed that the prevalence of serogroups O:4 and O:9 of all grouped isolates was estimated to be approximately equal at 44.6% and 45.5%, respectively. There was a similar prevalence of *Salmonella* Typhimurium (O:4) at 40.2% and *Salmonella* Enteritidis (O:9) at 41.4%. Some regional variation in serogroup and serovar distribution was identified with O:4 isolates accounting for the largest proportion of isolates in Africa, driven by the high prevalence of *Salmonella* Typhimurium. In contrast, serogroup O:9 accounted for the largest proportion of isolates in Europe, driven by both *Salmonella* Enteritidis and *Salmonella* Dublin. In addition, there was a higher prevalence of serogroup O:4 and a lower prevalence of serogroup O:9 in LMICs compared to HICs.

Our data align with a previous systematic review through 2014 that focused on Africa and reported that *Salmonella* Typhimurium and *Salmonella* Enteritidis were the most prevalent serovars causing NTS invasive disease, accounting for more than 90% of isolates [10]. Of articles included in our systematic review, UN regions that provided 20 or more studies included the Africa, Asia, and Europe with the majority originating from Africa. Data from Oceania was limited to one article documenting just three isolates. Notably, UN subregions lacking data included Northern Africa, Central Asia, Micronesia, Melanesia, and Polynesia (Supplementary Figure S2). In some countries, the lack of data could be partially explained by low incidence of NTS disease. Additionally, HICs are more likely to report data on NTS invasive disease in routine surveillance systems reports that were out of scope for this study. Another contributor to limited data for some regions was that *Salmonella* isolates were not always reported to the serogroup or serovar level. It is likely that for some countries NTS invasive disease was prevalent, but such data were not available due to a lack of strain typing.

Our meta-analysis showed substantial heterogeneity across the included articles. This was expected as articles from a range of UN regions and settings including both hospital-based and community-based studies were eligible. In addition, the large proportion of articles that were classified as high risk of bias could have contributed to the heterogeneity. Most of the included articles were assessed to be at a high risk of bias for microbiology primarily due to incomplete reporting of microbiological methods. In addition, for one-third of the articles, the method used for *Salmonella* serotyping was unclear. We suggest that the reporting of microbiology methods should be improved. This should include details of the serotyping methods used and participation in external quality assurance programmes. When agglutination testing is used, details of the battery of anti-sera available in the testing laboratory should be reported. For *in silico* serotyping, reporting of the bioinformatics pipeline, and version used, would assist with interpretation.

For vaccine products currently in development, we have provided estimates for potential coverage based on estimated serogroup and serovar prevalence. Assuming that vaccines would provide cross-protection for serovars in the same serogroup, live-attenuated *Salmonella* Typhimurium vaccine products would have the potential to cover 63.3% of all serogrouped isolates. If no within serogroup cross-protection is assumed, the target of *Salmonella* Typhimurium would cover 59.7% of all serotyped isolates. Assuming within serogroup cross-protection, the *Salmonella* Typhimurium and *Salmonella* Enteritidis Generalised Modules for Membrane Antigens (GMMA) vaccine and the trivalent *Salmonella* glycoconjugate vaccine targeting both *Salmonella* Typhimurium and *Salmonella* Enteritidis have the potential to cover 93.8% of all isolates classified to the serogroup level. The addition of coverage for serogroup O:7 would increase coverage by 4.4%. If no within serogroup cross-protection is assumed, vaccines targeting *Salmonella* Typhimurium and *Salmonella* Enteritidis alone would cover approximately 87.1% of isolates. Further addition of *Salmonella* Dublin or *Salmonella* Heidelberg would increase the coverage by 2%. In addition, our data inform decisions on the incremental benefit of adding serogroups or serovars to improve coverage of strains associated with invasive disease. Since vaccines vary considerably in the range of antigens used and in likely level of protection, assumptions about coverage and cross-protection should be interpreted with caution. In addition, our coverage estimates would likely vary by region and over time since regional and temporal variation of serogroups and serovars was observed.

In the Democratic Republic of the Congo, the emergence of a *Salmonella* Typhimurium O:5-negative variant has been reported, a strain lacking O:5 specificity [33, 34]. This O:5-negative variant represented 37% of all *Salmonella* Typhimurium in the study by Tack *et al.* For vaccine products based on O:4 antigens, such as the *Salmonella* glycoconjugate vaccine, the emergence of this O:5-negative variant may not influence their potential coverage. Nevertheless, the fact that O antigens could be lost over time poses a risk to vaccine coverage [35].

We searched multiple databases without restriction on time period, language, country, or the number of isolates. To our knowledge, this study is the first to provide global prevalence data for invasive NTS by serogroup and serovar by region. However, some limitations merit comment. First, this study was based on a previous publication that focused on complications and mortality of NTS invasive disease. Therefore, the search strategy included the terms ‘complications’ and ‘mortality’, which were not necessarily in the study scope and may have resulted in missing articles relevant to the research question. To be as comprehensive as possible, all articles that passed title and abstract review for the initial study were re-screened for eligibility for the present study. Second, we acknowledge that a minority of isolates were not further classified, and that this group included both isolates that were typed to the serogroup or serovar level and those reported as ‘other’ by the original authors, as well as isolates that were not typed. Additional data from the authors of eligible papers were not requested. To ensure that estimates were based on known serogroups and serovars, isolates were excluded if not classified to the serogroup or serovar level. As such, our study might overestimate the prevalence of serogroups or serovars for which anti-sera are more commonly available. For instance, in the African region, the proportion of serovars with a ranking of sixth and higher was <1%, suggesting that the serovars ranked first through fifth were dominant. Also, in Africa, the range of anti-sera might have been more limited than in other regions [36, 37]. Third, our analysis focused on the prevalence of serogroups and serovars and did not consider sequence types such as *Salmonella* Typhimurium ST313 and *Salmonella* Enteritidis ST11. While this likely has limited relevance for vaccine development, we were unable to evaluate the distribution of major sequence types. Fourth, while we appreciate the importance of host risk factors other than HIV, such as malnutrition and malaria, for NTS invasive disease, these factors were not reported sufficiently frequently to be evaluated in our study.

## Conclusion

Of global serogrouped NTS from normally sterile sites, serogroups O:4 and O:9 accounted for 90% isolates, and of global serotyped NTS from normally sterile sites *Salmonella* Typhimurium and Enteritidis together accounted for 75% of isolates. Some geographic and temporal variation in serogroup and serovar distribution was observed. Among UN regions, serogroup O:4 had the highest prevalence in the African region, driven by *Salmonella* Typhimurium, and likewise serogroup O:9 in the European region, driven by *Salmonella* Enteritidis and *Salmonella* Dublin. Vaccine development strategies that cover both serogroups O:4 and O:9, or *Salmonella* Typhimurium and *Salmonella* Enteritidis, have the potential to prevent the majority of NTS invasive disease.

## Supporting information

Hagedoorn et al. supplementary material Hagedoorn et al. supplementary material

## Data Availability

Data underlying the study results are available at https://doi.org/10.7910/DVN/AYFRX1. For additional data enquiries please contact the corresponding author john.crump@otago.ac.nz.

## Abbreviations

GMMA: Generalised Modules for Membrane Antigens -
NTS: Non-typhoidal *Salmonella*
PROSPERO: Prospective Register of Systematic Reviews
UN: United Nations

## Supplementary material

For supplementary material accompanying this paper visit http://doi.org/10.1017/S0950268823001693. click here to view supplementary material
