# Supplementary material for: Prevalence and distribution of non-typhoidal Salmonella enterica serogroups and serovars isolated from normally sterile sites: A global systematic review
Source: Epidemiol Infect. 2023 Oct 18;152:e4. doi: 10.1017/S0950268823001693 (PMC10789991; doi:10.1017/S0950268823001693)
Supplement: Hagedoorn et al. supplementary material [file S0950268823001693sup001.docx]

# Epidemiology and Infection

Prevalence and distribution of non-typhoidal *Salmonella* *enterica* serogroups and serovars isolated from normally sterile sites: a global systematic review

Nienke N. Hagedoorn^1^, Shruti Murthy^1^, Megan Birkhold^2^, Christian S. Marchello^1^, John A. Crump^1*^, on behalf of the Vacc-iNTS Consortium Collaborators

## Supplementary Table S1 – Serogroup classification based on serogroups and serovars identified in the systematic review on prevalence of serogroups and serovars of non-typhoidal *Salmonella enterica* isolated from normally sterile sites, global, 1941-2019 [1]

| ***Salmonella* serogroup** | ***Salmonella* serovar** | | | | | | | | | | | | | |
| --- | --- | --- | --- | --- | --- | --- | --- | --- | --- | --- | --- | --- | --- | --- |
| **O:2 (A)** | Group A |  |  |  |  |  |  |  |  |  |  |  |  |  |
| **O:4 (B)** | Abony | Agona | Agama | Brancaster | Brandenburg | Bredeney | Chester | Coeln | Copenhagen | Derby | Haifa | Hato | Heidelberg | Java |
|  | Kaapstad | Kiambu | Kisangani | Reading | Saintpaul | Sandiego | Schwarzengrund | Stanley | Stanleyville | Typhimurium | Wien | Group B |  |  |
| **O:7 (C1)** | Augustenborg | Bareilly | Braenderup | Choleraesuis | Colindale | Galiema | Hartford | Infantis | Irumu | Isangi | Livingstone | Mbandaka | Montevideo | Norwich |
|  | Oakland | Ohio | Oranienburg | Othmarschen | Rissen | Tennessee | Thompson | Virchow | Group C |  |  |  |  |  |
| **O:8 (C2-C3)** | Albany | Bardo | Blockley | Bovismorbificans | Corvallis | Hadar | Kentucky | Kottbus | Litchfield | Manhattan | Muenchen | Newport | Tshiongwe | Group C2 |
| **O:9 (D1)** | Berta | Blegdam | Dublin | Enteritidis | Javiana | Miami | Napoli | Panama | Group D |  |  |  |  |  |
| **O:9,46 (D2)** | Group D2 |  |  |  |  |  |  |  |  |  |  |  |  |  |
| **O:3,10 (E1)** | Amsterdam | Anatum | Give | London | Muenster | Uganda | Weltevreden |  |  |  |  |  |  |  |
| **O:1,3,19 (E4)** | Krefeld | Senftenberg |  |  |  |  |  |  |  |  |  |  |  |  |
| **O:11 (F)** | Chandans | Rubislaw | Senegal |  |  |  |  |  |  |  |  |  |  |  |
| **O:13 (G)** | Havana | Kedougou | Mississippi | Poona | Raus | Telelkebir |  |  |  |  |  |  |  |  |
| **O:16 (I)** | Gaminara | Hvittingfoss |  |  |  |  |  |  |  |  |  |  |  |  |
| **O:18 (K)** | Cerro |  |  |  |  |  |  |  |  |  |  |  |  |  |
| **O:21 (L)** | Minnesota |  |  |  |  |  |  |  |  |  |  |  |  |  |
| **O:28 (M)** | Kibusi | Telaviv | Umbilo |  |  |  |  |  |  |  |  |  |  |  |
| **O:30 (N)** | Matopeni | Urbana |  |  |  |  |  |  |  |  |  |  |  |  |
| **O:35 (O)** | Adelaide |  |  |  |  |  |  |  |  |  |  |  |  |  |
| **O:38 (P)** | Freetown |  |  |  |  |  |  |  |  |  |  |  |  |  |
| **O:40 (R)** | Johannesburg |  |  |  |  |  |  |  |  |  |  |  |  |  |
| **Undesignated** | Aviana | Vitiki |  |  |  |  |  |  |  |  |  |  |  |  |

## Supplementary Table S2 – Bias assessment methods for the systematic review on prevalence of serogroups and serovars of non-typhoidal *Salmonella enterica* isolated from normally sterile sites, adopted from Marchello, 2022 [2]

| **Grading** | **Definition** |  |  |
| --- | --- | --- | --- |
| L | Low risk of bias |  |  |
| M | Moderate risk of bias |  |  |
| H | High risk of bias |  |  |
| U | Unknown or unavailable to assess bias |  |  |
|  |  |  |  |
| **Domain question** | **Explanation** | **Low risk definition** | **High risk definition** |
| Study design (L/H/U) | Type of study design used to ascertain NTS deaths and complications. | Active population- or household-based surveillance, prospective observational | Passive surveillance, retrospective studies, case-control, medical or laboratory records review |
| Study setting (L/H/U) | Hospital/inpatient or community/outpatient | Community or outpatient based recruitment | Admitted or inpatient hospital-based recruitment |
| Patient selection (L/H/U) | Were specific populations targeted or were all patients were eligible for recruitment | Described a systematic collection of blood culture among eligible patients | Targeted a population (e.g. children, only adults, or groups such as HIV only) or non-NTS clinical syndrome (e.g. respiratory) |
| Final year of data collection (L/M/H) | More confidence in newer studies having more robust recruitment and analytical methods | Data collection from 2010 through 2020 | Data collection 2000 and older |
| Microbiology methods (L/H/U) | Were microbiology methods fully presented, including blood culture volume and contamination. Clearly defined how infection was attributed to NTS and not *Salmonella* Typhi or Paratyphi A, B, or C. | Appropriate microbiological methods presented for ruling out typhoidal serotypes. Described blood culture volume and contamination. | Unclear or unconventional microbiological methods. No information on blood culture volumes and contamination. |
|  |  |  |  |
| **Domain subtotal** |  |  |  |
| 0 of 3 H risk | Low risk |  |  |
| 1 of 3 H risk | Moderate risk |  |  |
| >1 H risk | High risk |  |  |
|  |  |  |  |
| **Overall risk of bias** |  |  |  |
| Both subtotals low risk | Low risk |  |  |
| 1 of 2 high risk | Moderate risk |  |  |
| Both subtotals high risk | High risk |  |  |

## Supplementary Table S3 - Details of included articles identified in the global systematic review on prevalence of serogroups and serovars of non-typhoidal *Salmonella enterica* isolated from normally sterile sites, 1941 to 2019 (82 articles)

| **First author, publication year (reference)** | **Country** | **UN region** | **UN sub region** | **Study locality** | **Setting** | **Sterile site cultured** | **Date start data collection** | **Date end data collection** | **Methods serovar typing** | **Age group** | **Overall bias** | **Number of different reported serovars** | **Number of different serogroups** | **Total reported isolates** |
| --- | --- | --- | --- | --- | --- | --- | --- | --- | --- | --- | --- | --- | --- | --- |
| Albert, 2019 [3] | Kuwait | Asia | Western Asia | Kuwait City | Hospital-based | blood | 01/04/2013 | 31/05/2016 | Mlst | Mixed ages | H | 16 | 9 | 61 |
| Angrist, 1946 [4] | United States of America | Americas | Northern America | New York | Hospital-based | blood | 01/01/1941 | 31/12/1944 | Cross-agglutination test | Mixed ages | H | 3 | 3 | 11 |
| Appiah, 2021 [5] | Uganda | Africa | Eastern Africa | Apac, Tororo, Arua, Kabale, Jinja, and Mubende | Hospital-based | blood | 01/07/2016 | 31/01/2019 | Unclear | Children only (<=15y) | M | 2 | 3 | 31 |
| Asseva, 2012 [6] | Bulgaria | Europe | Eastern Europe | n/a | National surveillance | blood; csf; deep tissue; other | 01/01/2005 | 31/12/2010 | Cross-agglutination test | Mixed ages | H | 5 | 5 | 33 |
| Aubry, 1992 [7] | Burundi | Africa | Eastern Africa | Bujumbura | Hospital-based | blood | 01/01/1991 | 31/12/1991 | Unclear | Adults only (>15y) | H | 2 | 3 | 54 |
| Barrios, 2017 [8] | Uruguay | Americas | South America | Montevideo | Hospital-based | blood | 01/01/2005 | 31/12/2010 | Unclear | Children only (<=15y) | H | 4 | 4 | 10 |
| Bassa, 1989 [9] | Spain | Europe | Southern Europe | Palma de Mallorca | Hospital-based | blood | 01/01/1979 | 30/04/1988 | Unclear | Mixed ages | H | 2 | 3 | 43 |
| Berkowitz, 1984 [10] | South Africa | Africa | Southern Africa | Soweto | Hospital-based | blood | 01/01/1982 | 31/12/1982 | Unclear | Children only (<=15y) | M | 10 | 5 | 47 |
| Blomberg, 2007 [11] | Tanzania | Africa | Eastern Africa | Dar es Salaam | Hospital-based | blood | 01/08/2001 | 31/08/2002 | Unclear | Children only (<=15y) | M | 3 | 4 | 37 |
| Brent, 2006 [12] | Kenya | Africa | Eastern Africa | Kilifi | Hospital-based | blood | 01/08/1998 | 31/07/2002 | Cross-agglutination test | Children only (<=15y) | H | 2 | 3 | 166 |
| Cheesbrough, 1997 [13] | Democratic Republic of the Congo | Africa | Middle Africa | Western | Hospital-based | blood | 01/01/1990 | 31/12/1992 | Cross-agglutination test | Children only (<=15y) | M | 2 | 3 | 33 |
| Chen, 1999 [14] | Taiwan | Asia | Eastern Asia | Southern | Hospital-based | blood | 01/01/1991 | 31/12/1996 | Cross-agglutination test | Mixed ages | H | 3 | 3 | 39 |
| Chen, 2012 [15] | Taiwan | Asia | Eastern Asia | South | Hospital-based | blood | 01/01/1996 | 31/12/2008 | Cross-agglutination test | Adults only (>15y) | H | 1 | 4 | 151 |
| Chu, 2014 [16] | Taiwan | Asia | Eastern Asia | Chang Gung | Hospital-based | blood | 01/01/2004 | 31/12/2011 | Unclear | Children only (<=15y) | H | 0 | 2 | 1 |
| Ciftci, 2004 [17] | Turkey | Asia | Western Asia | Ankara | Hospital-based | blood | 01/01/1993 | 31/12/2002 | Unclear | Children only (<=15y) | H | 3 | 4 | 29 |
| Cisneros-Herreros, 2005 [18] | Spain | Europe | Southern Europe | Sevilla | Hospital-based | blood | 01/06/2001 | 17/04/2002 | Unclear | Adults only (>15y) | H | 1 | 2 | 4 |
| Cisterna, 2001 [19] | Spain | Europe | Southern Europe | Bilbao | Hospital-based | blood | 01/01/1994 | 30/09/2001 | Unclear | Mixed ages | H | 1 | 2 | 82 |
| Dhanoa, 2009 [20] | Malaysia | Asia | South-eastern Asia | George Town | Hospital-based | blood | 01/07/2002 | 31/07/2006 | Cross-agglutination test | Mixed ages | H | 7 | 6 | 52 |
| Falay, 2016 [21] | Democratic Republic of the Congo | Africa | Middle Africa | Oriental Province | Hospital-based | blood | 01/05/2009 | 31/05/2014 | Cross-agglutination test | Children only (<=15y) | H | 3 | 3 | 113 |
| Feasey, 2015 [22] | Malawi | Africa | Eastern Africa | Blantyre | Hospital-based | blood | 01/01/1998 | 31/12/2014 | Cross-agglutination test | Mixed ages | M | 2 | 3 | 10139 |
| Galanakis, 2007 [23] | Greece | Europe | Southern Europe | Heraklion | Hospital-based | blood; synovial fluid | 01/01/1993 | 31/12/2002 | Cross-agglutination test | Children only (<=15y) | H | 5 | 5 | 14 |
| Gbadoe, 2008 [24] | Togo | Africa | Western Africa | Lomé | Hospital-based | blood | 01/01/1995 | 31/12/2004 | Cross-agglutination test | Children only (<=15y) | H | 3 | 3 | 60 |
| Georgilis, 1997 [25] | Greece | Europe | Southern Europe | Athens | Hospital-based | blood | 01/01/1987 | 31/12/1996 | Cross-agglutination test | Adults only (>15y) | H | 5 | 4 | 28 |
| Gilks, 1990 [26] | Kenya | Africa | Eastern Africa | Nairobi | Hospital-based | blood | 30/11/1988 | 15/05/1989 | Unclear | Adults only (>15y) | M | 2 | 3 | 12 |
| Glaser, 1985 [27] | United States of America | Americas | Northern America | New York | Hospital-based | blood | 01/10/1981 | 15/02/1984 | Unclear | Adults only (>15y) | H | 3 | 4 | 16 |
| Gonzalez-Hevia, 1990 [28] | Spain | Europe | Southern Europe | Aviles | Laboratory-based | blood; other | 01/01/1984 | 31/12/1987 | Cross-agglutination test | Mixed ages | H | 5 | 4 | 14 |
| Gowda, 2017 [29] | Australia | Oceania | Australia and New Zealand | Queensland | Hospital-based | blood; csf | 01/01/2005 | 01/01/2016 | Unclear | Children only (<=15y) | H | 1 | 2 | 3 |
| Grant, 1998 [30] | Côte d’Ivoire | Africa | Western Africa | Abidjan | Hospital-based | blood | 04/12/1995 | 18/03/1996 | Unclear | Adults only (>15y) | H | 1 | 2 | 11 |
| Guiraud, 2017 [31] | Burkina Faso | Africa | Western Africa | Nanoro | Hospital-based | blood | 13/05/2013 | 12/05/2014 | Cross-agglutination test | Children only (<=15y) | M | 4 | 4 | 67 |
| Gundogdu, 2017 [32] | Türkiye | Asia | Western Asia | Kayseri | Laboratory-based | blood | 01/04/2013 | 31/01/2016 | Cross-agglutination test | Mixed ages | M | 2 | 3 | 7 |
| Habib, 2004 [33] | Singapore | Asia | South-eastern Asia | Singapore | Hospital-based | blood | 01/04/2001 | 31/03/2003 | Unclear | Mixed ages | H | 2 | 3 | 26 |
| Harich, 2017 [34] | India | Asia | Southern Asia | Kerala | Hospital-based | blood; synovial fluid | 01/08/2011 | 31/07/2013 | Cross-agglutination test | Mixed ages | H | 1 | 2 | 13 |
| Henderson, 1947 [35] | Panama | Americas | Central America | Ancon | Laboratory-based | blood; csf; deep tissue; other | 01/01/1942 | 31/12/1946 | Cross-agglutination test | Mixed ages | H | 6 | 5 | 13 |
| Hsu, 2003 [36] | Taiwan | Asia | Eastern Asia | Taipei | Hospital-based | blood | 01/09/1995 | 30/09/2001 | Cross-agglutination test | Mixed ages | H | 0 | 5 | 93 |
| Huang, 2004 [37] | Taiwan | Asia | Eastern Asia | Kaohsiung | Hospital-based | blood | 01/01/1996 | 31/01/2002 | Cross-agglutination test | Children only (<=15y) | H | 2 | 3 | 45 |
| Ispahani, 2000 [38] | United Kingdom | Europe | Northern Europe | Nottingham | Hospital-based | blood; csf; synovial fluid; other | 01/01/1980 | 31/12/1997 | Cross-agglutination test | Mixed ages | H | 16 | 6 | 104 |
| Jones, 2008 [39] | United States of America | Americas | Northern America | 5-10 states | National surveillance | blood; csf; synovial fluid; other | 01/01/1996 | 31/12/2006 | Unclear | Mixed ages | H | 53 | 14 | 2524 |
| Kariuki, 2006 [40] | Kenya | Africa | Eastern Africa | Nairobi | Hospital-based | blood | 01/03/2002 | 31/05/2005 | Cross-agglutination test | Children only (<=15y) | H | 2 | 3 | 198 |
| Kassa-Kelembho, 2003 [41] | Central African Republic | Africa | Middle Africa | Bangui | Hospital-based | blood | 01/04/1999 | 31/07/1999 | Cross-agglutination test | Adults only (>15y) | M | 3 | 4 | 22 |
| Katiyo, 2019 [42] | United Kingdom | Europe | Northern Europe | England | National surveillance | blood | 01/01/2004 | 31/12/2015 | Combination of methods | Mixed ages | H | 20 | 6 | 2484 |
| Keddy, 2017 [43] | South Africa | Africa | Southern Africa | Gauteng Province | Laboratory-based | other | 01/01/2003 | 31/12/2013 | Unclear | Mixed ages | H | 4 | 4 | 4347 |
| Kedzierska, 2008 [44] | Poland | Europe | Eastern Europe | Cracow | Hospital-based | blood; deep tissue; other | 01/01/2000 | 31/12/2006 | Cross-agglutination test | Adults only (>15y) | H | 4 | 4 | 30 |
| Koch, 2011 [45] | Denmark | Europe | Northern Europe | North Jutland, Aarhus, Funen | Laboratory-based | blood | 01/01/1999 | 31/12/2008 | Cross-agglutination test | Mixed ages | M | 18 | 7 | 313 |
| Lee, 2005 [46] | Malaysia | Asia | South-eastern Asia | Kuala Lumpur | Hospital-based | csf; deep tissue; synovial fluid; other | 01/01/1993 | 31/12/2002 | Cross-agglutination test | Mixed ages | H | 6 | 7 | 12 |
| Lepage, 1989 [47] | Rwanda | Africa | Eastern Africa | Kigali | Hospital-based | blood | 15/10/1986 | 14/12/1986 | Cross-agglutination test | Children only (<=15y) | H | 2 | 3 | 16 |
| Lepage, 1987 [48] | Rwanda | Africa | Eastern Africa | Kigali | Community-based | blood | 16/10/1984 | 15/10/1985 | Cross-agglutination test | Children only (<=15y) | H | 3 | 3 | 36 |
| Lester, 1991 [49] | Denmark | Europe | Northern Europe | Copenhagen | Laboratory-based | blood | 01/01/1984 | 31/12/1988 | Unclear | Mixed ages | H | 6 | 4 | 168 |
| Maltha, 2014 [50] | Burkina Faso | Africa | Western Africa | Nanoro | Hospital-based | blood; csf | 01/07/2012 | 31/07/2013 | Unclear | Children only (<=15y) | M | 3 | 4 | 21 |
| Mandal, 1988 [51] | United Kingdom - England | Europe | Northern Europe | Manchester | Hospital-based | blood | 01/01/1975 | 31/12/1983 | Unclear | Mixed ages | H | 13 | 6 | 108 |
| Mandomando, 2015 [52] | Mozambique | Africa | Eastern Africa | Manhiça | Hospital-based | blood | 01/01/2001 | 31/12/2014 | Combination of methods | Children only (<=15y) | M | 16 | 8 | 620 |
| Matas, 1995 [53] | Spain | Europe | Southern Europe | Barcelona | Hospital-based | blood | 01/01/1991 | 31/12/1991 | Cross-agglutination test | Mixed ages | H | 0 | 2 | 66 |
| Mohan, 2019 [54] | Malaysia | Asia | South-eastern Asia | Bintulu | Hospital-based | blood | 01/01/2011 | 31/12/2016 | Cross-agglutination test | Children only (<=15y) | H | 9 | 7 | 38 |
| Muthumbi, 2015 [55] | Kenya | Africa | Eastern Africa | Kilifi | Hospital-based | blood; csf | 01/08/1998 | 31/12/2014 | Cross-agglutination test | Mixed ages | H | 2 | 3 | 351 |
| Nathoo, 1996 [56] | Zimbabwe | Africa | Eastern Africa | Harare | Hospital-based | blood | 01/06/1993 | 31/12/1994 | Cross-agglutination test | Children only (<=15y) | M | 1 | 2 | 10 |
| Nelson, 1982 [57] | United States of America | Americas | Northern America | St. Louis | Hospital-based | blood | 01/01/1975 | 31/01/1981 | Cross-agglutination test | Children only (<=15y) | H | 4 | 3 | 5 |
| Noriega, 1994 [58] | Belgium | Europe | Western Europe | Brussels | Hospital-based | blood; deep tissue; pleural fluid | 01/01/1975 | 31/12/1990 | Unclear | Adults only (>15y) | H | 4 | 3 | 29 |
| Papaevangelou, 2004 [59] | Greece | Europe | Southern Europe | Athens | Hospital-based | blood | 01/06/1990 | 31/05/2002 | Cross-agglutination test | Children only (<=15y) | H | 5 | 5 | 119 |
| Patra, 2018 [60] | India | Asia | Southern Asia | Manipal | Hospital-based | blood | 01/01/2012 | 31/12/2016 | Cross-agglutination test | Mixed ages | H | 1 | 2 | 40 |
| Phoba, 2014 [61] | Democratic Republic of the Congo | Africa | Middle Africa | Bwamanda | Hospital-based | blood | 01/11/2011 | 31/05/2012 | Cross-agglutination test | Children only (<=15y) | M | 2 | 3 | 85 |
| Phu Huong Lan, 2016 [62] | Vietnam | Asia | South-eastern Asia | Ho Chi Minh City | Hospital-based | blood | 01/01/2008 | 30/06/2013 | Combination of methods | Mixed ages | M | 3 | 4 | 89 |
| Phuong, 2017 [63] | Lao People's Democratic Republic | Asia | South-eastern Asia | Vientiane | Hospital-based | blood | 01/01/2006 | 31/12/2012 | Combination of methods | Mixed ages | M | 5 | 4 | 63 |
| Preveden, 2001 [64] | Serbia | Europe | Southern Europe | Novi Sad | Hospital-based | blood | 01/01/1991 | 31/12/1998 | Unclear | Mixed ages | H | 2 | 3 | 12 |
| Preziosi, 2015 [65] | Mozambique | Africa | Eastern Africa | Maputo | Hospital-based | blood | 01/09/2011 | 30/03/2014 | Cross-agglutination test | Adults only (>15y) | H | 2 | 3 | 10 |
| Prignet, 1993 [66] | France | Europe | Southern Europe | Toulon | Hospital-based | blood | 01/01/1972 | 31/12/1991 | Unclear | Mixed ages | H | 8 | 4 | 22 |
| Ramos, 1996 [67] | Spain | Europe | Southern Europe | Madrid | Hospital-based | blood | 01/01/1960 | 31/12/1992 | Cross-agglutination test | Mixed ages | H | 2 | 3 | 92 |
| Raucher, 1983 [68] | United States | Americas | Northern America | New York | Hospital-based | blood | 01/01/1981 | 31/12/1981 | Cross-agglutination test | Children only (<=15y) | H | 5 | 3 | 7 |
| Roberts, 1993 [69] | Canada | Americas | Northern America | Vancouver | Hospital-based | blood | 01/01/1980 | 31/01/1992 | Cross-agglutination test | Adults only (>15y) | H | 11 | 5 | 21 |
| Secmeer, 1995 [70] | Turkey | Asia | Western Asia | Ankara | Hospital-based | blood; bone marrow; csf | 01/08/1982 | 30/09/1992 | Unclear | Children only (<=15y) | H | 2 | 3 | 75 |
| Seydi, 2005 [71] | Senegal | Africa | Western Africa | Dakar | Hospital-based | blood | 01/01/1996 | 31/12/2003 | Cross-agglutination test | Mixed ages | H | 2 | 3 | 49 |
| Shimoni, 1999 [72] | Israel | Asia | Western Asia | Petach Tikva | Hospital-based | blood | 01/01/1987 | 31/12/1996 | Cross-agglutination test | Mixed ages | H | 7 | 5 | 73 |
| Sirinavin, 1999 [73] | Thailand | Asia | South-eastern Asia | Bangkok | Hospital-based | blood; csf; pleural fluid; synovial fluid; other | 01/01/1978 | 31/12/1994 | Cross-agglutination test | Children only (<=15y) | H | 4 | 5 | 172 |
| Sow, 1994 [74] | Senegal | Africa | Western Africa | Dakar | Hospital-based | blood | 01/01/1985 | 31/12/1989 | Unclear | Children only (<=15y) | H | 7 | 6 | 16 |
| Still, 2020 [75] | Mali | Africa | Western Africa | Bamako | Hospital-based & Outpatient | blood; csf; synovial fluid; other | 01/06/2002 | 31/12/2018 | Combination of methods | Children only (<=15y) | M | 3 | 3 | 682 |
| Tabu, 2012 [76] | Kenya | Africa | Eastern Africa | Asembo (rural) & Kibera (urban) | Hospital-based | blood | 01/10/2006 | 30/09/2009 | Cross-agglutination test | Mixed ages | H | 3 | 3 | 67 |
| Tack, 2020 [77] | Democratic Republic of the Congo | Africa | Middle Africa | Kisantu | Hospital-based | blood | 01/01/2015 | 31/10/2017 | Cross-agglutination test | Mixed ages | M | 5 | 6 | 896 |
| Vandenberg, 2010 [78] | Democratic Republic of the Congo | Africa | Middle Africa | Kivu Province | Hospital-based | blood | 01/01/2002 | 31/12/2006 | Cross-agglutination test | Children only (<=15y) | H | 4 | 4 | 191 |
| Vlieghe, 2012 [79] | Cambodia | Asia | South-eastern Asia | Phnom Penh | Hospital-based | blood | 01/07/2007 | 30/06/2011 | Cross-agglutination test | Mixed ages | M | 5 | 5 | 50 |
| Walsh, 2000 [80] | Malawi | Africa | Eastern Africa | Blantyre | Hospital-based | blood | 01/09/1996 | 31/08/1997 | Cross-agglutination test | Children only (<=15y) | M | 2 | 3 | 140 |
| Wilkens, 1997 [81] | Ghana | Africa | Western Africa | Accra | Hospital-based | blood | 07/12/1993 | 07/03/1994 | Cross-agglutination test | Children only (<=15y) | M | 4 | 4 | 17 |
| Yen, 2009 [82] | Taiwan | Asia | Eastern Asia | Taipei | Hospital-based | blood | 01/01/2004 | 31/12/2006 | Cross-agglutination test | Adults only (>15y) | H | 3 | 4 | 71 |
| Yombi, 2015 [83] | Belgium | Europe | Western Europe | Brussels | Hospital-based | blood | 01/01/2007 | 31/12/2012 | Unclear | Mixed ages | H | 2 | 3 | 20 |
| Zaidi, 1999 [84] | United States | Americas | Northern America | Boston, MA | Hospital-based | blood | 01/01/1979 | 31/12/1995 | Unclear | Children only (<=15y) | H | 5 | 4 | 138 |

Legend Supplementary Table S3: CSF, cerebrospinal fluid; MLST, multilocus sequence typing

## Supplementary Table S4 – Descriptive characteristics by article and by isolates identified in the global systematic review on prevalence of serogroups and serovars of non-typhoidal *Salmonella* *enterica* isolated from normally sterile sites, 1941 to 2019

|  | **Included articles, n=82** | **Reported isolates, n=26,280** |
| --- | --- | --- |
|  | N (%) | N (%) |
| **Data collection duration in years, median (IQR)** | 6 (3-10) | - |
| **Number of serogroups, median (IQR)** | 3 (2-4) | - |
| **Number of serovars, median (IQR)** | 3 (2-5) | - |
| **UN regions and UN subregions** |  |  |
| Africa | 31 (37.8) | 18544 (70.6) |
| Eastern Africa | 15 (19.3) | 11887 (45.2) |
| Middle Africa | 6 (7.3) | 1340 (5.1) |
| Northern Africa | - | - |
| Southern Africa | 2 (2.4) | 4394 (16.7) |
| Western Africa | 8 (9.8) | 923 (3.5) |
| The Americas | 9 (10.9) | 2745 (10.4) |
| Caribbean | - | - |
| Central America | 1 (1.2) | 13 (0) |
| Northern America | 7 (8.5) | 2722 (10.4) |
| South America | 1 (1.2) | 10 (0) |
| Asia | 21 (25.6) | 1203 (4.6) |
| Central Asia | - | - |
| Eastern Asia | 6 (7.3) | 403 (1.5) |
| South-eastern Asia | 8 (9.8) | 502 (1.9) |
| Southern Asia | 2 (2.4) | 53 (0.2) |
| Western Asia | 5 (6.1) | 245 (0.9) |
| Europe | 20 (24.4) | 3785 (14.4) |
| Eastern Europe | 2 (2.4) | 63 (0.2) |
| Northern Europe | 5 (6.1) | 3177 (12.1) |
| Southern Europe | 11 (13.4) | 496 (1.9) |
| Western Europe | 2 (2.4) | 49 (0.2) |
| Oceania | 1 (1.2) | 3 (0) |
| Australia and New Zealand | 1 (1.2) | 3 (0) |
| Micronesia | - | - |
| Melanesia | - | - |
| Polynesia | - | - |
| **Income group** |  |  |
| High-income country | 37 (45.1) | 7048 (26.8) |
| Lower- and middle-income country | 45 (54.9) | 19229 (73.2) |
| **Setting** |  |  |
| Community-based | 1 (1.2) | 36 (0.1) |
| Hospital-based | 71 (86.6) | 15659 (59.6) |
| Laboratory-based | 6 (7.3) | 4862 (18.5) |
| National surveillance | 3 (3.7) | 5041 (19.2) |
| Combined | 1 (1.2) | 682 (2.6) |
| **Age group** |  |  |
| Adults only | 13 (15.8) | 459 (1.8) |
| Children only | 33 (40.2) | 3242 (12.3) |
| Mixed ages | 36 (43.9) | 22579 (58.9) |
| **Sterile site cultured** |  |  |
| Blood | 80 (97.6) | - |
| Bone marrow | 1 (1.2) | - |
| Cerebrospinal fluid | 11 (13.4) | - |
| Deep tissue | 5 (6.1) |  |
| Pleural fluid | 2 (2.4) | - |
| Synovial fluid | 7 (8.5) | - |
| Other normally sterile sites* | 10 (12.2) | - |
| **Methods serovar typing** |  |  |
| Agglutination testing | 49 (59.7) |  |
| Combination of methods | 5 (6.1) |  |
| Multilocus sequence typing | 1 (1.2) |  |
| Unclear | 27 (32.9) |  |

Supplementary Table S4: IQR, interquartile range; UN, united nations

*Other normally sterile sites include bile, bronchoalveolar lavage, normally sterile body site undefined, peritoneal fluid, subdural fluid, urine collected with catheter, wound secretion or pus.

## Supplementary Table S5 - The proportion of non-typhoidal *Salmonella enterica* isolates by serogroup for all serogrouped isolates identified in the global systematic review on prevalence of serogroups and serovars of non-typhoidal *Salmonella enterica* from normally sterile sites, 1941 to 2019 (24,253 isolates)

| ***Salmonella s*erogroup** | **N isolates (% of serogrouped isolates)** | |
| --- | --- | --- |
| O:4 (B) | 15345 | (63.3) |
| O:9 (D1) | 7386 | (30.5) |
| O:7 (C1) | 1063 | (4.4) |
| O:8 (C2-C3) | 250 | (1) |
| O:13 (G) | 104 | (0.4) |
| O:3,10 (E1) | 37 | (0.2) |
| O:1,3,19 (E4) | 21 | (0.1) |
| O:30 (N) | 14 | (0.1) |
| O:11 (F) | 8 | (0) |
| O:18 (K) | 5 | (0) |
| O:40 (R) | 4 | (0) |
| O:16 (I) | 4 | (0) |
| O:21 (L) | 3 | (0) |
| O:28 (M) | 3 | (0) |
| O:35 (O) | 3 | (0) |
| O:2 (A) | 1 | (0) |
| O:38 (P) | 1 | (0) |
| O:9,46 (D2) | 1 | (0) |

## Supplementary Table S6 – The proportion of non-typhoidal *Salmonella enterica* isolates of serotyped isolates in the global systematic review on prevalence of serogroups and serovars of non-typhoidal Salmonella enterica isolated from normally sterile sites, 1941 to 2019 (23,971 isolates)

| ***Salmonella* serovar** | **N (%) of serotyped isolates)** |
| --- | --- |
| Typhimurium | 14314 (59.7%) |
| Enteritidis | 6561 (27.4%) |
| Dublin | 524 (2.2%) |
| Heidelberg | 473 (2%) |
| Choleraesuis | 225 (0.9%) |
| Virchow | 218 (0.9%) |
| Isangi | 176 (0.7%) |
| Oranienburg | 138 (0.6%) |
| Newport | 121 (0.5%) |
| Poona | 95 (0.4%) |
| Montevideo | 88 (0.4%) |
| Panama | 82 (0.3%) |
| Infantis | 79 (0.3%) |
| Java | 75 (0.3%) |
| Saintpaul | 71 (0.3%) |
| Javiana | 69 (0.3%) |
| Schwarzengrund | 59 (0.2%) |
| Stanley | 58 (0.2%) |
| Corvallis | 35 (0.1%) |
| Hadar | 33 (0.1%) |
| Brandenburg | 33 (0.1%) |
| Sandiego | 31 (0.1%) |
| Agona | 29 (0.1%) |
| Bovismorbificans | 27 (0.1%) |
| Braenderup | 23 (0.1%) |
| Chester | 21 (0.1%) |
| Berta | 17 (0.1%) |
| Thompson | 17 (0.1%) |
| Colindale | 16 (0.1%) |
| Bareilly | 15 (0.1%) |
| Krefeld | 15 (0.1%) |
| Urbana | 13 (0.1%) |
| Muenchen | 12 (0.1%) |
| Muenster | 12 (0%) |
| Ohio | 11 (0%) |
| Derby | 11 (0%) |
| Anatum | 11 (0%) |
| Reading | 10 (0%) |
| Bredeney | 9 (0%) |
| Mbandaka | 8 (0%) |
| Tennessee | 7 (0%) |
| Rubislaw | 6 (0%) |
| Senftenberg | 6 (0%) |
| Uganda | 6 (0%) |
| Cerro | 5 (0%) |
| Copenhagen | 5 (0%) |
| Kentucky | 5 (0%) |
| Mississippi | 4 (0%) |
| Johannesburg | 4 (0%) |
| Give | 4 (0%) |
| Livingstone | 4 (0%) |
| Litchfield | 4 (0%) |
| Hartford | 3 (0%) |
| Blockley | 3 (0%) |
| Gaminara | 3 (0%) |
| Aviana | 3 (0%) |
| Blegdam | 3 (0%) |
| Haifa | 3 (0%) |
| Minnesota | 3 (0%) |
| Kiambu | 3 (0%) |
| Adelaide | 3 (0%) |
| Bardo | 2 (0%) |
| Abony | 2 (0%) |
| Albany | 2 (0%) |
| Irumu | 2 (0%) |
| Kedougou | 2 (0%) |
| Kisangani | 2 (0%) |
| Napoli | 2 (0%) |
| Telelkebir | 2 (0%) |
| Tshiongwe | 2 (0%) |
| Weltevreden | 2 (0%) |
| Miami | 2 (0%) |
| Manhattan | 1 (0%) |
| Agama | 1 (0%) |
| Amsterdam | 1 (0%) |
| Brancaster | 1 (0%) |
| Chandans | 1 (0%) |
| Coeln | 1 (0%) |
| Freetown | 1 (0%) |
| Galiema | 1 (0%) |
| Hato | 1 (0%) |
| Havana | 1 (0%) |
| Hvittingfoss | 1 (0%) |
| Kaapstad | 1 (0%) |
| Kibusi | 1 (0%) |
| Kottbus | 1 (0%) |
| London | 1 (0%) |
| Matopeni | 1 (0%) |
| Oakland | 1 (0%) |
| Othmarschen | 1 (0%) |
| Rissen | 1 (0%) |
| Senegal | 1 (0%) |
| Stanleyville | 1 (0%) |
| Telaviv | 1 (0%) |
| Umbilo | 1 (0%) |
| Vitiki | 1 (0%) |
| Wien | 1 (0%) |

## Supplementary Table S7 – The proportion of non-typhoidal *Salmonella enterica* serogroups and serovars by HIV infection status of participants, isolated from normally sterile sites, global, 1941-2019 (10 articles)

|  | **HIV-infected participants** | **HIV-uninfected participants** | **P-value of Chi-square test** |
| --- | --- | --- | --- |
|  | **n (%)** | **n (%)** |  |
| **Serogroup** | n=1,743 | n=257 |  |
| O:4 | 1174 (67.4) | 89 (34.6) | <0.01 |
| O:9 | 559 (32.1) | 157 (61.1) | <0.01 |
| Other | 10 (0.6) | 11 (4.3) | <0.01 |
|  |  |  |  |
| **Serovar** | n=1,743 | n=258* |  |
| *S.* Typhimurium | 1174 (67.4) | 88 (34.1) | <0.01 |
| *S.* Enteritidis | 493 (28.3) | 147 (57.0) | <0.01 |
| Other | 76 (4.4) | 23 (8.9) | <0.01 |

*1 isolate was serotyped, but not serogrouped (*S.* Vitiki)

## Supplementary Figure S1 – PRISMA flowchart of study selection process for the global systematic review on prevalence of serogroups and serovars of non-typhoidal *Salmonella* *enterica* from normally sterile sites, 1941 to 2019

Supplementary Figure S1: *Multiple reasons for exclusion possible

## Supplementary Figure S2 - Global distribution of number of articles per country identified in the global systematic review on prevalence of serogroups and serovars of non-typhoidal *Salmonella enterica* isolated from normally sterile sites, 1941 to 2019 (82 articles)

## Supplementary Figure S3 – Bias assessment of the global systematic review on prevalence of serogroups and serovars of non-typhoidal *Salmonella enterica* isolated from normally sterile sites, 1941 to 2019 (82 articles)

__

## Supplementary Figure S4 - Global distribution of serogrouped isolates by serogroup by decade, global systematic review on prevalence of serogroups and serovars of non-typhoidal Salmonella enterica isolated from normally sterile sites, 1941 to 2019 (82 articles, 24,253 isolates)

## Supplementary Figure S5 - Prevalence of non-typhoidal *Salmonella enterica* serogroups from normally steriles, by UN region, 1941-2019 (82 articles, 24,253 isolates)

Legend Supplementary Figure S5: *Other serogroups with <10 cases.


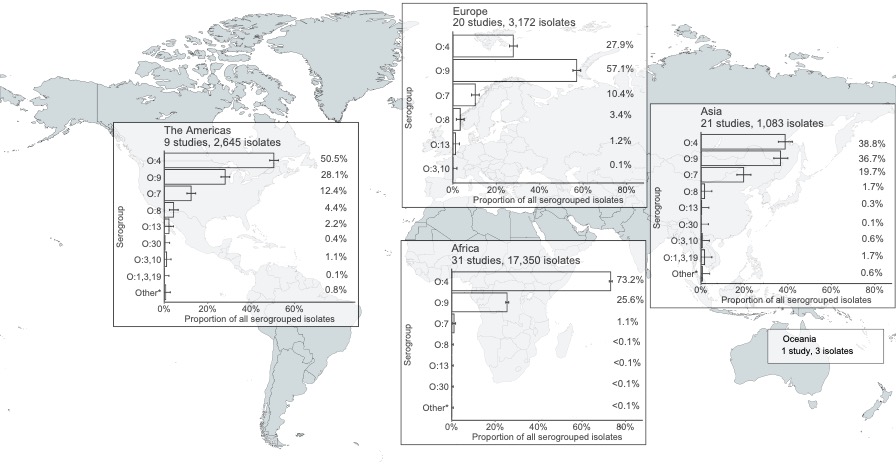


## Supplementary Figure S6 - Forest plot of meta-analysis of prevalence of non-typhoidal *Salmonella enterica* from normally sterile sites of all serogrouped isolates: serogroup O:4, serogroup O:9, and other serogroups, 1941-2019 (82 articles, 24,253 isolates)

## Supplementary Figure S7 - Forest plot of meta-analysis of prevalence of non-typhoidal *Salmonella enterica* from normally sterile sites of all serogrouped isolates: serogroup O:4, serogroup O:9, and other serogroups per UN region, 1941-2019 (82 articles, 24,253 isolates)

## Supplementary Figure S8 - Forest plot of meta-analysis of prevalence of non-typhoidal *Salmonella enterica* from normally sterile sites of all serogrouped isolates: serogroup O:4, serogroup O:9, and other serogroups per age groups, 1941-2019 (82 articles, 24,253 isolates)

## Supplementary Figure S9 - Forest plot of meta-analysis of prevalence of non-typhoidal *Salmonella enterica* serovar Typhimurium, *Salmonella enterica* serovar Enteritidis and other serovars from normally sterile sites, 1941-2019 (79 articles, 23,971 isolates)

# References

1. **Grimont P, *et al.*** (2007) Antigenic Formulae of the *Salmonella* serovars, (9th ed.) Paris: WHO Collaborating Centre for Reference and Research on *Salmonella*. *Institute Pasteur.***,** 1-166.

2. **Marchello CS, *et al.*** (2022) Complications and mortality of non-typhoidal *Salmonella* invasive disease: a global systematic review and meta-analysis. *Lancet Infectious Diseases* **22**(5)**,** 692-705. <https://doi.org/10.1016/S1473-3099(21)00615-0>.

3. **Albert MJ, *et al.*** (2019) Non-typhoidal *Salmonella* blood stream infection in Kuwait: Clinical and microbiological characteristics. *PLoS Negl Trop Dis* **13**(4)**,** e0007293. <https://doi.org/10.1371/journal.pntd.0007293>.

4. **Angrist A, *et al.*** (1946) Bacteriologic, clinical and pathologic experience with 86 sporadic cases of *Salmonella* infection. *American Journal of the Medical Sciences* **212**(3)**,** 336-346.

5. **Appiah GD, *et al.*** (2021) *Salmonella* Bloodstream Infections in Hospitalized Children with Acute Febrile Illness-Uganda, 2016-2019. *Am J Trop Med Hyg* **105**(1)**,** 37-46. <https://doi.org/10.4269/ajtmh.20-1453>.

6. **Asseva G, *et al.*** (2012) Systemic and extraintestinal forms of human infection due to non-typhoid salmonellae in Bulgaria, 2005-2010. *European Journal of Clinical Microbiology and Infectious Diseases* **31**(11)**,** 3217-3221.

7. **Aubry P, *et al.*** (1992) [Bacteremia caused by non-typhoid Salmonellas during an infection by the human immunodeficiency virus (HIV) in the African adult]. *Médecine tropicale : revue du Corps de santé colonial* **52**(4)**,** 447-450.

8. **Barrios P, *et al.*** (2017) [A five-year experience with zoonotic *Salmonella* at a pediatric reference centre]. *Revista chilena de infectología* **34**(4)**,** 359-364.

9. **Bassa A, *et al.*** (1989) Non-typhi *Salmonella* bacteraemia. *Infection* **17**(5)**,** 290-293. <https://doi.org/10.1007/BF01650710>.

10. **Berkowitz FE** (1984) Bacteremia in hospitalized Black South African children. A one-year study emphasizing nosocomial bacteremia and bacteremia in severely malnourished children. *American Journal of Diseases of Children* **138**(6)**,** 551-556.

11. **Blomberg B, *et al.*** (2007) Antimicrobial resistance predicts death in Tanzanian children with bloodstream infections: a prospective cohort study. *BMC Infect Dis* **7,** 43. <https://doi.org/10.1186/1471-2334-7-43>.

12. **Brent AJ, *et al.*** (2006) *Salmonella* bacteremia in Kenyan children. *Pediatr Infect Dis J* **25**(3)**,** 230-236. <https://doi.org/10.1097/01.inf.0000202066.02212.ff>.

13. **Cheesbrough JS, *et al.*** (1997) Clinical definition for invasive *Salmonella* infection in African children. *The Pediatric Infectious Disease Journal* **16**(3)**,** 277-283.

14. **Chen YH, *et al.*** (1999) Epidemiological study of human salmonellosis during 1991-1996 in southern Taiwan. *Kaohsiung Journal of Medical Sciences* **15**(3)**,** 127-136.

15. **Chen PL, *et al.*** (2012) Non-typhoidal *Salmonella* bacteraemia in elderly patients: An increased risk for endovascular infections, osteomyelitis and mortality. *Epidemiology and Infection* **140**(11)**,** 2037-2044.

16. **Chu SM, *et al.*** (2014) Neurological complications after neonatal bacteremia: The clinical characteristics, risk factors, and outcomes. *PLOS ONE* **9**(11)**,** e105294.

17. **Ciftci E, *et al.*** (2004) *Salmonella* bacteraemia in Turkish children: 37 cases seen in a university hospital between 1993 and 2002. *Ann Trop Paediatr* **24**(1)**,** 75-80. <https://doi.org/10.1179/027249304225013295>.

18. **Cisneros-Herreros JM, *et al.*** (2005) Blood cultures in the emergency department. *Enfermedades Infecciosas y Microbiologia Clinica* **23**(3)**,** 135-139.

19. **Cisterna R, *et al.*** (2001) Community-acquired bacteremia. *Revista Espanola de Quimioterapia* **14**(4)**,** 369-382.

20. **Dhanoa A, *et al.*** (2009) Non-typhoidal *Salmonella* bacteraemia: Epidemiology, clinical characteristics and its' association with severe immunosuppression. *Annals of Clinical Microbiology and Antimicrobials* **8,** 15.

21. **Falay D, *et al.*** (2016) Microbiological, clinical and molecular findings of non-typhoidal *Salmonella* bloodstream infections associated with malaria, Oriental Province, Democratic Republic of the Congo. *BMC Infect Dis* **16,** 271. <https://doi.org/10.1186/s12879-016-1604-1>.

22. **Feasey NA, *et al.*** (2015) Three epidemics of invasive multidrug-resistant *Salmonella* bloodstream infection in Blantyre, Malawi, 1998-2014. *Clinical Infectious Diseases* **61,** S363-S371.

23. **Galanakis E, *et al.*** (2007) Invasive non-typhoidal salmonellosis in immunocompetent infants and children. *Int J Infect Dis* **11**(1)**,** 36-39. <https://doi.org/10.1016/j.ijid.2005.09.004>.

24. **Gbadoe AD, *et al.*** (2008) Pediatric salmonellosis at the Tokoin's teaching hospital, Lome (Togo). *Medecine et Maladies Infectieuses* **38**(1)**,** 8-11.

25. **Georgilis K, *et al.*** (1997) Clinical and microbiologic aspects of nontyphi *Salmonella* bacteremia in adults. *Infectious Diseases in Clinical Practice* **6**(6)**,** 396-400.

26. **Gilks CF, *et al.*** (1990) Life-threatening bacteraemia in HIV-1 seropositive adults admitted to hospital in Nairobi, Kenya. *Lancet* **336**(8714)**,** 545-549. <https://doi.org/10.1016/0140-6736(90)92096-z>.

27. **Glaser JB, *et al.*** (1985) Recurrent *Salmonella* typhimurium bacteremia associated with the acquired immunodeficiency syndrome. *Ann Intern Med* **102**(2)**,** 189-193. <https://doi.org/10.7326/0003-4819-102-2-189>.

28. **Gonzalez-Hevia MA, *et al.*** (1990) [*Salmonella* enterica in the Aviles health district (Asturias). Microbiological, clinical, and epidemiological aspects]. *Salmonella enterica en el area sanitaria de Aviles (Asturias). Aspectos microbiologicos, clinicos y epidemiologicos.* **8**(3)**,** 144-147.

29. **Gowda H, *et al.*** (2017) Late-onset Neonatal Sepsis - A 10-year Review from North Queensland, Australia. *Pediatric Infectious Disease Journal* **36**(9)**,** 883-888.

30. **Grant AD, *et al.*** (1998) Spectrum of disease among HIV-infected adults hospitalised in a respiratory medicine unit in Abidjan, Cote d'Ivoire. *International Journal of Tuberculosis and Lung Disease* **2**(11)**,** 926-934.

31. **Guiraud I, *et al.*** (2017) Population-based incidence, seasonality and serotype distribution of invasive salmonellosis among children in Nanoro, rural Burkina Faso. *PLOS ONE* **12**(7)**,** e0178577. <https://doi.org/10.1371/journal.pone.0178577>.

32. **Gundogdu A, *et al.*** (2017) Distribution and Antimicrobial Resistance of *Salmonella* Serovars Isolated in Kayseri Region. *Klimik Journal* **30**(1)**,** 22-26.

33. **Habib AG** (2004) A clinical audit of presentation and outcome of *Salmonella* septicaemia. *Ann Acad Med Singap* **33**(6)**,** 749-753.

34. **Harich, *et al.*** (2017) Antimicrobial susceptibility profile, treatment outcome and serotype distribution of clinical isolates of *Salmonella* enterica subspecies enterica: A 2-year study from Kerala, South India. *Infection and Drug Resistance* **10,** 97-101.

35. **Henderson LL** (1947) *Salmonella* infections in Panama - review of 219 consecutive hospital cases occurring in the 5 year period 1942-1946. *American Journal of Tropical Medicine* **27**(5)**,** 643-655.

36. **Hsu RB, *et al.*** (2003) Risk factors for primary bacteremia and endovascular infection in patients without acquired immunodeficiency syndrome who have nontyphoid salmonellosis. *Clinical Infectious Diseases* **36**(7)**,** 829-834.

37. **Huang IF, *et al.*** (2004) Nontyphoid salmonellosis in taiwan children: clinical manifestations, outcome and antibiotic resistance. *J Pediatr Gastroenterol Nutr* **38**(5)**,** 518-523. <https://doi.org/10.1097/00005176-200405000-00011>.

38. **Ispahani P, *et al.*** (2000) Enteric fever and other extraintestinal salmonellosis in University Hospital, Nottingham, UK, between 1980 and 1997. *European Journal of Clinical Microbiology and Infectious Diseases* **19**(9)**,** 679-687.

39. **Jones TF, *et al.*** (2008) Salmonellosis outcomes differ substantially by serotype. *Journal of Infectious Diseases* **198**(1)**,** 109-114.

40. **Kariuki S, *et al.*** (2006) Characterisation of community acquired non-typhoidal *Salmonella* from bacteraemia and diarrhoeal infections in children admitted to hospital in Nairobi, Kenya. *BMC Microbiology* **6,** 101.

41. **Kassa-Kelembho E, *et al.*** (2003) Bacteremia in adults admitted to the Department of Medicine of Bangui Community Hospital (Central African Republic). *Acta Tropica* **89**(1)**,** 67-72.

42. **Katiyo S, *et al.*** (2019) Epidemiology and Outcomes of Nontyphoidal *Salmonella* Bacteremias from England, 2004 to 2015. *J Clin Microbiol* **57**(1). <https://doi.org/10.1128/JCM.01189-18>.

43. **Keddy KH, *et al.*** (2017) Clinical and microbiological features of invasive nontyphoidal *Salmonella* associated with HIV-infected patients, Gauteng Province, South Africa. *Medicine (Baltimore)* **96**(13)**,** e6448. <https://doi.org/10.1097/MD.0000000000006448>.

44. **Kedzierska J, *et al.*** (2008) Clinical presentation of extraintestinal infections caused by non-typhoid *Salmonella* serotypes among patients at the University Hospital in Cracow during an 7-year period. *Pol J Microbiol* **57**(1)**,** 41-47.

45. **Koch K, *et al.*** (2011) International travel and the risk of hospitalization with non-typhoidal *Salmonella* bacteremia. A Danish population-based cohort study, 1999-2008. *BMC Infectious Diseases* **11,** 277.

46. **Lee WS, *et al.*** (2005) Focal non-typhoidal *Salmonella* infections from a single center in Malaysia. *Southeast Asian J Trop Med Public Health* **36**(3)**,** 678-682.

47. **Lepage P, *et al.*** (1989) Bacteraemia as predictor of HIV infection in African children. *Acta Paediatrica Scandinavica* **78**(5)**,** 763-766.

48. **Lepage P, *et al.*** (1987) Community-acquired bacteraemia in African children. *Lancet* **1**(8548)**,** 1458-1461. <https://doi.org/10.1016/s0140-6736(87)92207-0>.

49. **Lester A, *et al.*** (1991) Non-typhoid *Salmonella* bacteraemia in Greater Copenhagen 1984 to 1988. *Eur J Clin Microbiol Infect Dis* **10**(6)**,** 486-490. <https://doi.org/10.1007/BF01963934>.

50. **Maltha J, *et al.*** (2014) Frequency of severe malaria and invasive bacterial infections among children admitted to a rural hospital in Burkina Faso. *PLOS ONE* **9**(2)**,** e89103. <https://doi.org/10.1371/journal.pone.0089103>.

51. **Mandal BK, *et al.*** (1988) Bacteraemia in salmonellosis: a 15 year retrospective study from a regional infectious diseases unit. *Bmj* **297**(6658)**,** 1242-1243. <https://doi.org/10.1136/bmj.297.6658.1242>.

52. **Mandomando I, *et al.*** (2015) Invasive *Salmonella* Infections Among Children From Rural Mozambique, 2001-2014. *Clin Infect Dis* **61 Suppl 4,** S339-345. <https://doi.org/10.1093/cid/civ712>.

53. **Matas Andreu L, *et al.*** (1995) Bacteremia in 13 general hospitals from Barcelona. A prospective study of 1674 episodes. *Enfermedades Infecciosas y Microbiologia Clinica* **13**(6)**,** 345-355.

54. **Mohan A, *et al.*** (2019) Invasive *Salmonella* infections among children in Bintulu, Sarawak, Malaysian Borneo: a 6-year retrospective review. *BMC Infect Dis* **19**(1)**,** 330. <https://doi.org/10.1186/s12879-019-3963-x>.

55. **Muthumbi E, *et al.*** (2015) Invasive Salmonellosis in Kilifi, Kenya. *Clin Infect Dis* **61 Suppl 4,** S290-301. <https://doi.org/10.1093/cid/civ737>.

56. **Nathoo KJ, *et al.*** (1996) Community-acquired bacteremia in human immunodeficiency virus-infected children in Harare, Zimbabwe. *Pediatric Infectious Disease Journal* **15**(12)**,** 1092-1097.

57. **Nelson SJ, *et al.*** (1982) *Salmonella* gastroenteritis in the first three months of life. A review of management and complications. *Clinical Pediatrics* **21**(12)**,** 709-712.

58. **Noriega LM, *et al.*** (1994) *Salmonella* infections in a cancer center. *Support Care Cancer* **2**(2)**,** 116-122. <https://doi.org/10.1007/BF00572093>.

59. **Papaevangelou V, *et al.*** (2004) *Salmonella* bacteraemia in a tertiary children's hospital. *Scandinavian Journal of Infectious Diseases* **36**(8)**,** 547-551.

60. **Patra S, *et al.*** (2018) Invasive nontyphoidal *Salmonella* disease in southern India: a 5-year experience from a tertiary care hospital. *Turk J Med Sci* **48**(5)**,** 1030-1035. <https://doi.org/10.3906/sag-1804-90>.

61. **Phoba MF, *et al.*** (2014) Epidemic increase in *Salmonella* bloodstream infection in children, Bwamanda, the Democratic Republic of Congo. *Eur J Clin Microbiol Infect Dis* **33**(1)**,** 79-87. <https://doi.org/10.1007/s10096-013-1931-8>.

62. **Phu Huong Lan N, *et al.*** (2016) Invasive Non-typhoidal *Salmonella* Infections in Asia: Clinical Observations, Disease Outcome and Dominant Serovars from an Infectious Disease Hospital in Vietnam. *PLoS Negl Trop Dis* **10**(8)**,** e0004857. <https://doi.org/10.1371/journal.pntd.0004857>.

63. **Phuong TLT, *et al.*** (2017) Non-typhoidal *Salmonella* serovars associated with invasive and non-invasive disease in the Lao People's Democratic Republic. *Transactions of the Royal Society of Tropical Medicine and Hygiene* **111**(9)**,** 418-424.

64. **Preveden T, *et al.*** (2001) [*Salmonella* bacteremia]. *Med Pregl* **54**(7-8)**,** 367-370.

65. **Preziosi M, *et al.*** (2015) A prospective observational study of bacteraemia in adults admitted to an urban Mozambican hospital. *South African Medical Journal* **105**(5)**,** 370-374.

66. **Prignet JM, *et al.*** (1993) Clinical features of non-intestinal non-typhoidal salmonellosis A review of forty-two cases and of the literature. *Semaine Des Hopitaux* **69**(16)**,** 465-474.

67. **Ramos JM, *et al.*** (1996) Classifying extraintestinal non-typhoid *Salmonella* infections. *QJM - Monthly Journal of the Association of Physicians* **89**(2)**,** 123-126.

68. **Raucher HS, *et al.*** (1983) Treatment of *Salmonella* gastroenteritis in infants. The significance of bacteremia. *Clin Pediatr (Phila)* **22**(9)**,** 601-604. <https://doi.org/10.1177/000992288302200901>.

69. **Roberts FJ** (1993) Nontyphoidal, nonparatyphoidal *Salmonella* septicemia in adults. *Eur J Clin Microbiol Infect Dis* **12**(3)**,** 205-208. <https://doi.org/10.1007/BF01967113>.

70. **Secmeer G, *et al.*** (1995) Prognostic factors in *Salmonella* typhimurium septicemia. A 10-year retrospective study. *Turk J Pediatr* **37**(3)**,** 229-233.

71. **Seydi M, *et al.*** (2005) [Current aspects of *Salmonella* bacteremia cases in the Ibrahima Diop Mar Infectious Diseases clinic, Fann National Hospital Center (Senegal)]. *Med Mal Infect* **35**(1)**,** 23-27. <https://doi.org/10.1016/j.medmal.2004.09.002>.

72. **Shimoni Z, *et al.*** (1999) Nontyphoid *Salmonella* bacteremia: age-related differences in clinical presentation, bacteriology, and outcome. *Clin Infect Dis* **28**(4)**,** 822-827. <https://doi.org/10.1086/515186>.

73. **Sirinavin S, *et al.*** (1999) Clinical and prognostic categorization of extraintestinal nontyphoidal *Salmonella* infections in infants and children. *Clin Infect Dis* **29**(5)**,** 1151-1156. <https://doi.org/10.1086/313469>.

74. **Sow D, *et al.*** (1994) Non typhoidic salmonellosis in the African pediatric population. *Dakar medical* **39**(1)**,** 51-55.

75. **Still WL, *et al.*** (2020) Surveillance for Invasive *Salmonella* Disease in Bamako, Mali, From 2002 to 2018. *Clin Infect Dis* **71**(Suppl 2)**,** S130-S140. <https://doi.org/10.1093/cid/ciaa482>.

76. **Tabu C, *et al.*** (2012) Differing burden and epidemiology of non-Typhi *Salmonella* bacteremia in rural and urban Kenya, 2006-2009. *PLOS ONE* **7**(2)**,** e31237. <https://doi.org/10.1371/journal.pone.0031237>.

77. **Tack B, *et al.*** (2020) Non-typhoidal *Salmonella* bloodstream infections in Kisantu, DR Congo: Emergence of O5-negative *Salmonella* Typhimurium and extensive drug resistance. *PLoS Negl Trop Dis* **14**(4)**,** e0008121. <https://doi.org/10.1371/journal.pntd.0008121>.

78. **Vandenberg O, *et al.*** (2010) Microbiologic and clinical features of *Salmonella* species isolated from bacteremic children in eastern Democratic Republic of Congo. *Pediatr Infect Dis J* **29**(6)**,** 504-510. <https://doi.org/10.1097/INF.0b013e3181cd615a>.

79. **Vlieghe ER, *et al.*** (2012) Azithromycin and ciprofloxacin resistance in *Salmonella* bloodstream infections in Cambodian adults. *PLoS Negl Trop Dis* **6**(12)**,** e1933. <https://doi.org/10.1371/journal.pntd.0001933>.

80. **Walsh AL, *et al.*** (2000) Bacteremia in febrile Malawian children: clinical and microbiologic features. *Pediatr Infect Dis J* **19**(4)**,** 312-318. <https://doi.org/10.1097/00006454-200004000-00010>.

81. **Wilkens J, *et al.*** (1997) *Salmonella* bloodstream infection in Ghanaian children. *Clinical Microbiology and Infection* **3**(6)**,** 616-620.

82. **Yen YF, *et al.*** (2009) Prognostic factors and clinical features of non-typhoid *Salmonella* bacteremia in adults. *J Chin Med Assoc* **72**(8)**,** 408-413. <https://doi.org/10.1016/S1726-4901(09)70397-1>.

83. **Yombi JC, *et al.*** (2015) Clinical features and outcome of typhoid fever and invasive non-typhoidal salmonellosis in a tertiary hospital in Belgium: analysis and review of the literature. *Acta Clin Belg* **70**(4)**,** 265-271. <https://doi.org/10.1179/2295333715Y.0000000016>.

84. **Zaidi E, *et al.*** (1999) Non-typhi *Salmonella* bacteremia in children. *Pediatr Infect Dis J* **18**(12)**,** 1073-1077. <https://doi.org/10.1097/00006454-199912000-00009>.
